# Review on Mining Robust Lactic Acid Bacteria for Next-Generation Silage Inoculants via Multi-Omics

**DOI:** 10.3390/life16010108

**Published:** 2026-01-12

**Authors:** Yanyan Liu, Mingxuan Zhao, Shanyao Zhong, Guoxin Wu, Fulin Yang, Jing Zhou

**Affiliations:** 1 College of Animal Sciences, Fujian Agriculture and Forestry University, Fuzhou 350002, China; 52406009026@fafu.edu.cn (Y.L.); m19863998873@163.com (M.Z.); 52406009015@fafu.edu.cn (S.Z.); wgx19981212@163.com (G.W.); 2 College of Resources and Environment, Fujian Agriculture and Forestry University, Fuzhou 350002, China

**Keywords:** silage, lactic acid bacteria, multi-omics technologies, microbial resources

## Abstract

Lactic acid bacteria (LAB), as the core microorganisms in silage fermentation, play a crucial role in improving silage quality and ensuring feed safety, making the screening, identification, and functional characterization of LAB strains a significant research focus. Researchers initially isolate and purify LAB from various samples, followed by identification through a combination of morphological, physiological, biochemical, and molecular biological methods. Systematic screening has been conducted to identify LAB strains tolerant to extreme environments (e.g., low temperature, high temperature, high salinity) and those possessing functional traits such as antimicrobial activity, antioxidant capacity, production of feruloyl esterase and bacteriocins, as well as cellulose degradation, yielding a series of notable findings. Furthermore, modern technologies, including microbiomics, metabolomics, metagenomics, and transcriptomics, have been employed to analyze the structure and functional potential of microbial communities, as well as metabolic dynamics during the ensiling process. The addition of superior LAB inoculants not only facilitates rapid acidification to reduce nutrient loss, inhibit harmful microorganisms, and improve fermentation quality and palatability but also demonstrates potential functions such as degrading mycotoxins, adsorbing heavy metals, and reducing methane emissions. However, its application efficacy is directly constrained by factors such as strain-crop specific interactions, high dependence on raw material conditions, limited functionality of bacterial strains, and relatively high application costs. In summary, the integration of multi-omics technologies with traditional methods, along with in-depth exploration of novel resources like phyllosphere endophytic LAB, will provide new directions for developing efficient and targeted LAB inoculants for silage.

## 1. Introduction

Silage is a high-quality roughage produced by chopping fresh forage and storing it under anaerobic conditions. In this process, epiphytic or inoculated lactic acid bacteria (LAB) ferment water-soluble carbohydrates, primarily into lactic acid, which rapidly lowers the pH. This acidic environment effectively suppresses the growth of spoilage microorganisms such as clostridia, yeasts, and molds. The resulting preserved feed is characterized by a soft texture, retained moisture, and enhanced palatability. Its role in providing stable, nutrient-rich feed year-round is vital for the sustainable development of the dairy and herbivorous livestock industries. The quality of silage is commonly evaluated based on key fermentation parameters: a pH below 4.2, a high lactic acid content relative to other fermentation acids, low ammonia-nitrogen concentration, and sustained aerobic stability upon exposure to air [1]. This study aims to review the isolation and identification of LAB for silage production. These microbes ferment water-soluble carbohydrate (WSC) into organic acids-mainly lactic acid, which quickly reduces the pH of the ensiled material. This acidic environment inhibits the activity of spoilage and pathogenic bacteria, enabling long-term preservation. LAB are the key microorganisms driving this fermentation. Their metabolic activity directly determines the efficiency and quality of the ensiling process. By rapidly converting sugars into lactic acid, LAB can lower the pH to below 4.0, effectively restraining harmful microbes such as *Clostridium* and molds, minimizing losses in protein and energy, and improving the palatability of the final feed product [2]. However, the natural ensiling process is susceptible to various factors such as forage variety, harvest timing, climatic conditions, and microbial composition, often leading to inconsistent fermentation quality and issues like spoilage or nutrient loss. Therefore, the application of exogenous, highly effective lactic acid bacteria inoculants has become a key technical approach to regulate fermentation and enhance silage quality.

In recent years, with continuous advances in LAB research, their application in silage has evolved beyond the role of simple “fermentation starters”. There is a growing shift toward screening “functional” strains with specific probiotic properties, such as the ability to degrade anti-nutritional factors, produce antimicrobial substances, adsorb mycotoxins and heavy metals, and enhance antioxidant activity [3]. These functional lactic acid bacteria not only enhance the fermentation quality of silage but also improve its nutritional value and additional benefits, with the potential to prevent animal diseases and promote health. The application of modern omics technologies, such as high-throughput sequencing, metabolomics, and metagenomics, enables researchers to systematically analyze the structure and function of the silage microecosystem and to uncover the mechanisms of lactic acid bacteria at the molecular level [4]. Furthermore, gene-editing technologies represented by CRISPR-Cas9 have provided new avenues for the targeted improvement and functional integration of lactic acid bacteria. This approach is expected to overcome the limitations of traditional strain screening and enable the design of “super” engineered strains that combine multiple desirable traits [1]. This study aims to elucidate the isolation and identification of LAB for silage production, promote the integration of multi-omics technologies with traditional methods, and explore novel resources such as phyllosphere endophytic lactic acid bacteria. The research seeks to provide a theoretical foundation and practical reference for developing superior LAB strains better suited for silage applications.

## 2. Application-Based Classification of LAB

### 2.1. Common Strains

A wide variety of LAB have been identified in nature, which are primarily distributed across 18 genera. Key genera include *Lactobacillus*, *Streptococcus*, and *Bifidobacterium*. Strains are selected based on specific application targets. In the food industry, the focus is on strains with rapid acid production and favorable flavor profiles. In the probiotic sector, priority is given to strains exhibiting robust tolerance to gastric acid and bile salts, along with strong mucosal adhesion capabilities [5].

### 2.2. Engineered Bacterial Strains

**Figure 1:**
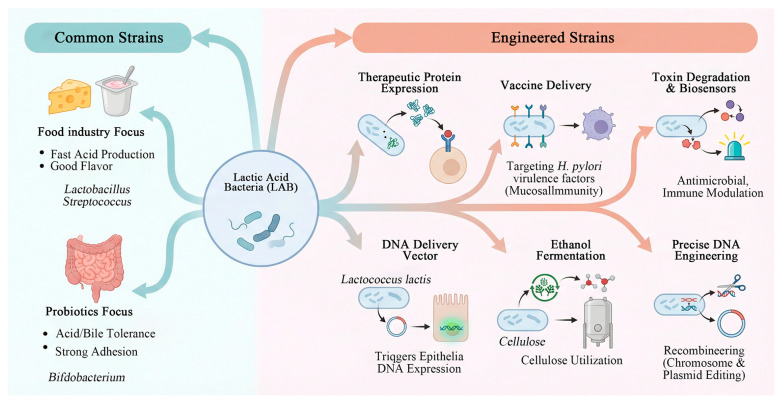
Classification of applications of lactic acid bacteria (this figure was created with Figdraw.com, and the copyright code is PROTT19ca4).

Engineered LAB have shown significant potential for applications in therapeutic protein expression, vaccine delivery, toxin degradation, and biosensing, with promising future prospects. Azevedo et al. [6] demonstrated that LAB such as *Lactococcus lactis* can serve as DNA delivery vectors, capable of inducing epithelial cells to express DNA both in vitro and in vivo. Zhang et al. [7] described a strategy targeting four virulence factors, which provides mucosal immunity against *Helicobacter pylori* infection. Van Pijkeren et al. [8] introduced innovative techniques such as recombinase-mediated genetic engineering (recombineering), enabling precise DNA manipulation of both chromosomal and plasmid elements in lactic acid bacteria (Figure 1).

### 2.3. Two Fermentation Methods of Lactic Acid Bacteria

The fundamental difference between homofermentative and heterofermentative lactic acid bacteria lies in their glucose metabolic pathways and end products. In silage applications, the two types are often used in combination; homofermentative bacteria are responsible for rapidly initiating fermentation and preserving nutrients, while heterofermentative bacteria enhance long-term preservation stability, creating a complementary advantage [9] (Table 1).

**Table 1:** Comparison of homofermentative and heterofermentative lactic acid bacteria.

	Homotypic Lactic Acid Bacteria	Heterotypic Fermented Lactic Acid Bacteria
Goals and Efficiency	Rapidly lower the pH. Ensure successful initial fermentation of silage, inhibit spoilage bacteria, and maximize the preservation of nutrients.	Efficiently produces acetic acid and other antibacterial substances. Enhances the aerobic stability of silage, inhibits the growth of yeast and mold, and prevents heating and spoilage.
Advantage	1. Fast fermentation rate 2. High lactic acid yield; the final pH is usually lower, ensuring more reliable preservation. 3. Minimal dry matter loss 4. Good palatability	1. The produced acetic acid and 1,2-propylene glycol, among other potent inhibitors, can effectively prevent secondary fermentation. 2. Acetic acid can also effectively inhibit Clostridia, which cause protein spoilage and butyric acid production.
Limitations	1. Poor aerobic stability 2. Sensitive to moisture 3. Potential for over-fermentation	1. Low fermentation efficiency 2. Significant dry matter loss. 3. Potential impact on palatability

## 3. Isolation and Purification of Lactic Acid Bacteria

### 3.1. Sources of Sample Collection

Samples for isolating lactic acid bacteria are obtained from diverse sources, commonly including silage and traditional fermented foods. Duan et al. [10] collected corn silage samples from pastures and dairy farms across northwestern regions, Yang et al. [11] gathered samples from pickled vegetables in various areas of Meishan City, and Liu et al. [12] obtained samples from pickle brine, goose intestines, duck intestines, chicken crops, commercially available yogurt, and other materials for strain isolation, purification, and identification.

### 3.2. Isolation and Purification

An appropriate amount of the sample was mixed with sterile saline or phosphate-buffered solution and thoroughly homogenized using a stomacher to prepare the sample extract [13]. To enrich strains with specific tolerance, selective culture media were subsequently employed. For instance, Lv et al. [14] screened acid-and bile-tolerant lactic acid bacteria by supplementing MRS (De Man, Rogosa and Sharpe) medium with 0.2–0.5% bile salts and adjusting the pH to an acidic range. Alternatively, after enrichment in liquid culture, serial 10-fold dilutions were performed, and the dilutions were plated onto solid isolation media for anaerobic incubation. Suspected lactic acid bacterial colonies were selected based on morphological characteristics, purified, and reserved for further identification [15].

### 3.3. Identification of Lactic Acid Bacteria

#### 3.3.1. Morphological Observation

In the morphological identification of LAB, colony characteristics serve as important diagnostic criteria. Typically, LAB are Gram-positive, non-spore-forming, non-motile, and exhibit either rod-shaped or coccoid morphologies, a profile consistent with findings reported by Duan et al. [10]. Qi et al. [16] reported that LAB strains isolated from pickled vegetables often formed colonies that were milky-white, circular, and smooth with a moist surface. Additionally, Niu et al. [17] noted that some strains capable of producing *Exopolysaccharides* (EPS) developed colonies with a mucoid or stringy appearance.

#### 3.3.2. Physiological and Biochemical Characterization

Physiological and biochemical characterization serves as a critical reference in the identification of LAB. The vast majority of LAB are catalase-negative, a key feature that distinguishes them from many other Gram-positive bacteria [18]. For instance, Xie et al. [19] reported that isolated LAB strains were catalase-negative and incapable of decomposing hydrogen peroxide to produce oxygen, in contrast to catalase-positive bacteria like staphylococci and micrococci. In their work to identify a strain of *Lactobacillus* producing alkaline phosphatase, Duan et al. [20] in identifying a strain of *Lactobacillus* producing alkaline phosphatase, referred to the results outlined in *Bergey’s Manual of Determinative Bacteriology* for *Lactobacillus* identification. The strain exhibited typical characteristics, including positive starch hydrolysis, negative catalase activity, and positive ammonia production from arginine. Based on these findings, it was preliminarily identified as *Lactobacillus rhamnosus*. Furthermore, Hu et al. [21] observed in radish kimchi that lactic acid bacteria could ferment a variety of sugars (e.g., glucose, lactose, sucrose, maltose, cellobiose) to produce acid, with some heterofermentative strains additionally capable of gas production.

#### 3.3.3. Molecular Biological Identification

**Figure 2:**
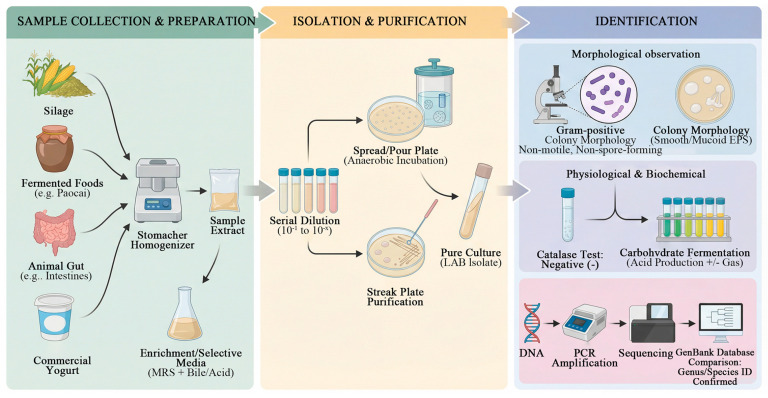
Isolation, purification, and identification of *lactic acid bacteria* (this figure was created with Figdraw.com, and the copyright code is OIIIR4fcff).

Sequencing and analysis of the 16S rRNA gene is currently the most accurate and universal molecular biology method for identifying lactic acid bacteria. Comparing sequencing results with databases such as GenBank enables precise identification at the genus or species level. This approach has been reliably used to identify lactic acid bacteria isolated from diverse sources, including traditional Indonesian fermented milk [22], Malaysian fermented foods [23], shrimp paste [24], and traditional shalgam juice [25], all through accurate 16S rRNA gene sequencing (Figure 2).

## 4. Probiotic Properties of Lactic Acid Bacteria

### 4.1. Screening of Lactic Acid Bacteria Under Extreme Environmental Conditions

#### 4.1.1. Screening and Characterization of Psychrotolerant Lactic Acid Bacteria from Plateau Regions

Numerous studies indicate that the alpine regions of China serve as a valuable resource for screening psychrotolerant lactic acid bacteria. Yang et al. [26] isolated and identified the cold-tolerant strain *Lactobacillus LCG3* from legume silage on the Tibetan Plateau. Using a restricted culture approach. Lin et al. [27] screened psychrotolerant lactic acid bacteria from the surface of oats on the Qinghai–Tibet Plateau, obtaining 18 cold-tolerant strains. Among these, OL54 was identified as a potential inoculant for low-temperature silage fermentation. Chen et al. [28] isolated psychrotolerant lactic acid bacteria from the pristine forest soil of Changbai Mountain, which demonstrated strong stress resistance, high acid production capacity, and effective in vitro antibacterial activity. *Lactobacillus C37*, *C34*, and *C4212* were selected as promising candidates for low-temperature silage fermentation during harsh winters in Northeast China. Xu et al. [29] screened cold-tolerant lactic acid bacteria from epiphytic microorganisms on several forage species native to the Qinghai–Tibet Plateau, including *Elymus nutans*, *Avena sativa* L., and *Koeleria tibetica*. A total of 148 lactic acid bacteria strains were isolated, of which four exhibited robust growth and rapid acidification capability both in MRS broth and green juice fermentation medium under low-temperature conditions.

#### 4.1.2. Screening and Application of Heat-Tolerant Lactic Acid Bacteria Under Tropical High-Temperature Conditions

To identify high-quality, heat-tolerant LAB suitable for tropical forage and to improve silage fermentation quality, Liu et al. [30] collected wild herbaceous plants such as *Saccharum arundinaceum* and *Microstegium vagans*, along with woody plants including *Leucaena leucocephala* and *Broussonetia papyrifera*, from different climatic regions of Hainan Island. Using fermentation broth with a pH ≤ 4 and OD ≥ 2 as preliminary screening criteria, strains with higher OD values were selected as dominant LAB isolates. These strains were identified as acid-tolerant, heat-resistant, and demonstrated excellent growth and acid production capabilities. They showed promising potential for silage application and exhibited effective inhibition against pathogenic bacteria, making them suitable candidates for developing silage additives in tropical regions. Additional studies have reported the isolation of *Lactobacillus delbrueckii L1* from fruit residue in Hainan, which exhibits strong heat tolerance and high acid-producing activity. Moreover, selective heat-tolerant LAB were successfully screened from fermented juice of tropical crops (FJLB) [31]. Subsequent inoculation of these LAB into Stylosanthes silage resulted in well-preserved forage with significantly improved fermentation quality, demonstrating the effectiveness of LAB in enhancing the preservation of tropical grasses.

#### 4.1.3. Screening Strategies for Salt Tolerance in Lactic Acid Bacteria in High-Salt Environments

The salt content in silage raw materials may inhibit the growth of LAB, making salt tolerance a critical criterion for evaluating LAB intended for silage use. Ideally, silage LAB should exhibit robust growth in a medium containing 3.0% NaCl. Papadopoulou et al. [32] employed adaptive evolution by gradually increasing salt concentrations in strains of *Lactiplantibacillus plantarum*, *Enterococcus faecium*, and *Pediococcus pentosaceus*. They found that the evolved *L. plantarum* strain produced 1.18 times more lactic acid than the wild-type, while the evolved E. faecium strain acquired the ability to produce lactic acid. In addition to adaptive evolution, direct isolation from high-salt environments or strain improvement through mutagenesis are also effective approaches. For instance, one study isolated salt-tolerant strains from traditionally fermented chili products and obtained a mutant, *L14U2-3*, through UV mutagenesis. This mutant demonstrated superior performance in acid production, acid tolerance, bile salt tolerance, and salt tolerance compared to the original strain [33]. Hao et al. [34] isolated a LAB strain designated *MMB-09* from farm brine. This strain exhibited excellent acid and salt tolerance, was identified as *L. plantarum*, and showed antimicrobial activity, making it suitable for use as a feed additive.

#### 4.1.4. Screening of Lactic Acid Bacteria with Antimicrobial Activity

Given that antimicrobial substances produced by LAB can effectively inhibit the growth of pathogenic and spoilage microorganisms in silage, their antimicrobial activity has become a key criterion for evaluating strain functionality. Previous studies have isolated two strains, F1 and T2, from native sources, which demonstrated significant antibacterial activity. Their inhibition zone diameters against pathogenic Escherichia coli and Salmonella reached 19/17 mm and 20/18 mm, respectively [35]. Similarly, Xiao et al. [36] screened 12 LAB strains from fermented fruit broth that exhibited inhibitory effects against intestinal pathogens (inhibition zone diameters ranging from 14 to 24 mm). Subsequent physiological, biochemical, and molecular identification confirmed that some of these strains belonged to *Lactobacillus paracasei*, thereby providing a basis for selecting strains with both antimicrobial function and probiotic potential.

### 4.2. Screening of Functional Lactic Acid Bacteria

Current research has shifted from merely selecting lactic acid bacteria that initiate fermentation to identifying “functional” strains with specific probiotic properties, such as possessing high antioxidant activity or having the ability to produce feruloyl esterase or bacteriocins. The scope of research on functional lactic acid bacteria has correspondingly expanded, extending from ecological interactions at the strain level to the molecular mechanisms underlying their functions.

#### 4.2.1. Deconstructors of Cellulose: Screening of Lactic Acid Bacteria Producing Feruloyl Esterase

The initial screening of LAB typically relies on selective media and phenotypic identification. For instance, plate screening methods often use ferulic acid ester analogs as substrates. The capacity of LAB to produce the target enzyme is preliminarily assessed by measuring the ratio of the hydrolysis zone surrounding a colony to the colony diameter. After incubation, isolates exhibiting clear hydrolysis zones were selected as candidates and further identified to species level via 16S rDNA sequencing. Lu et al. [37] combined selective plates with high-performance liquid chromatography (HPLC) for screening. Following the observation of clear zones on plates, HPLC was used to confirm enzyme activity, which reached up to 260 mU/L.

After obtaining candidate strains through phenotypic screening, molecular biology techniques are required for precise identification. Xu et al. [38] screened LAB with FAE activity from ensiled corn stover. Identification via 16S rRNA sequencing revealed *Lactobacillus amylovorus* as the strain exhibiting the most significant enzyme production. For more precise identification at the subspecies level, sequence analysis of housekeeping genes (e.g., *recA*, *pheS*, or *rpoA*) is necessary. In a study by Chen et al. [39] screening LAB from Bamei pig feces, in addition to 16S rDNA sequencing, *recA* gene amplification was utilized for the accurate identification of *Lactiplantibacillus plantarum* subspecies.

#### 4.2.2. Natural Defenders: Screening of Bacteriocin-Producing Lactic Acid Bacteria

Bacteriocins are ribosomally synthesized antimicrobial peptides or proteins, with LAB serving as important producers [40]. When screening for bacteriocin-producing LAB, after confirming bacteriocin production, the antimicrobial spectrum is typically determined to evaluate inhibitory effects against both Gram-positive and Gram-negative bacteria. Yi et al. [41] isolated *Lactobacillus pentosus DZ35* from kimchi and dry-cured meat products, which produces a bacteriocin with good thermal stability and maintained activity across a pH range of 2–11. Genomic sequencing and bioinformatics tools (e.g., *BAGEL4*) can further elucidate the bacteriocin gene clusters, clarifying genetic information related to their type, precursor peptides, modification enzymes, and associated transport proteins. Based on this approach, two novel bacteriocins, *pentocin DZ1* and *pentocin DZ2*, were identified in *Lactobacillus pentosus DZ35* [42]. Lv et al. [43] screened strain *ZW9* from soy sauce residue, whose antimicrobial substances remained stable at 40–100 °C and under UV irradiation, while retaining good activity within pH 2.0–6.0, demonstrating potential as a biological antimicrobial agent. Zhang et al. [44] isolated *Lactobacillus harbinensis P1-1* from traditional fermented foods; this strain exhibits both good gastrointestinal tolerance and the ability to inhibit Escherichia coli and Staphylococcus epidermidis, showing promise for development as an intestinal probiotic. In practical applications, Weinbrenner et al. [45] effectively suppressed over-acidifying lactic acid bacteria in yogurt by adding bacteriocins during the post-fermentation stage, delaying product post-acidification and enhancing quality stability.

#### 4.2.3. Guardians Against Oxidative Stress: Screening of Antioxidant Lactic Acid Bacteria

The screening of LAB with antioxidant activity is a multi-step and multi-index complex process. This procedure typically begins with in vitro chemical screening, where indicators such as the scavenging capacity against DPPH radicals, hydroxyl radicals, and superoxide anion radicals, as well as ferrous ion chelating ability and hydrogen peroxide tolerance, are measured to preliminarily identify strains with strong antioxidant potential. Subsequently, molecular biological identification (e.g., 16S rRNA sequencing) and cellular-level validation are conducted—for instance, using cell-free supernatants to assess their protective effects on oxidatively damaged cells. Studies have confirmed that some strains screened through this process not only exhibit tolerance to 2 mM hydrogen peroxide but also demonstrate hydrophobicity and auto-aggregation ability, suggesting promising probiotic potential [46].

Wang et al. [47] systematically evaluated the growth characteristics under high temperature, H_2_O_2_ tolerance, free radical scavenging capacity, reducing power, and exopolysaccharide production ability of five LAB strains isolated from Holstein cow milk, thereby comprehensively assessing their heat resistance and antioxidant capacity. Additionally, the potential intestinal colonization ability of the strains was verified through Caco-2 cell adhesion experiments. Furthermore, the antioxidant role of LAB is also reflected in their ability to enhance the bioactive compound content of substrates during fermentation. Wang et al. [48] found that fermentation of red bean milk by *Lactiplantibacillus plantarum Lp-G18* increased the total flavonoid and total polyphenol content by 1.2-fold and 2.2-fold, respectively, accompanied by a significant enhancement in DPPH and hydroxyl radical scavenging capacities.

#### 4.2.4. Screening of Biofilm-Producing Lactic Acid Bacteria

Lactic acid bacteria capable of forming biofilms are regarded as promising “fourth-generation probiotics”, as their biofilm structures can enhance resistance to harsh environmental conditions. Current research primarily focuses on two major directions. The first is the screening and evaluation of high biofilm-producing strains. For instance, Yang et al. [49] isolated and purified lactic acid bacteria from environmental samples and assessed their biofilm production using the crystal violet assay. The second direction can be seen in studies aiming to explore the practical functions and tolerance mechanisms of biofilms. Zhang et al. [50] compared planktonic and biofilm states and found that biofilms significantly improved the tolerance of lactic acid bacteria to acid, alkali, bile salts, and simulated gastrointestinal fluids, while also enhancing their antioxidant capacity. Olszewska et al. [51] employed plate counting and flow cytometry to analyze the impact of ethanol and acetic acid stress on the viability of biofilm-state *Lactiplantibacillus plantarum B1*, providing insights into the protective role of biofilms, and the results from both methods were consistent. *Lactiplantibacillus B1* forming biofilms exhibited significantly higher survival rates under ethanol and acetic acid stress. In complex systems such as silage, Li et al. [52] utilized metagenomics to reveal that although harmful bacteria dominated during the initial fermentation stage, the abundance of LAB genera carrying key carbohydrate-active enzyme genes-such as *Lactiplantibacillus* and *Pediococcus*-increased as fermentation progressed. This suggests that biofilm-forming strains may possess ecological advantages in colonization and functional performance.

#### 4.2.5. Screening of Lactic Acid Bacteria with Cellulose-Degrading Capacity

The ability of lactic acid bacteria to degrade cellulose is another important characteristic in silage fermentation. Such strains can directly break down cellulose in feed, thereby enhancing its nutritional value. Studies have confirmed that adding lactic acid bacteria with this function to silage, either alone or in combination with cellulase, can effectively improve fermentation quality. Li et al. [53] found that simultaneous addition of lactic acid bacteria and cellulase (or enzyme-producing microorganisms) significantly reduced the neutral detergent fiber and acid detergent fiber content in silage, while increasing lactic acid production and rumen degradation rate. Chen et al. [54] reported that composite addition of heat-tolerant *Lactiplantibacillus plantarum LP149* and *Acremonium cellulase* to hybrid napiergrass silage not only reduced fiber content but also optimized the microbial community, increased lactic and acetic acid levels, and comprehensively enhanced fermentation quality. Therefore, developing lactic acid bacteria possessing both feruloyl esterase and cellulase activities could provide an efficient and low-cost biological pretreatment strategy for silage production [55].

#### 4.2.6. Screening of Lactic Acid Bacteria for 1,2-Propanediol Production

Research by Berlowska Joanna et al. [56] demonstrates the potential of combining microbial fermentation with chemical catalysis. Their study utilized a simultaneous saccharification and fermentation (SSF) process, in which a mixed microbial consortium converted agricultural waste such as sugar beet pulp into lactic acid, followed by catalytic hydrogenation to produce propanediol. This approach provides a broader perspective for the development of lactic acid bacteria strains capable of producing 1,2-propanediol.

#### 4.2.7. Screening of Lactic Acid Bacteria for Pesticide Degradation

**Figure 3:**
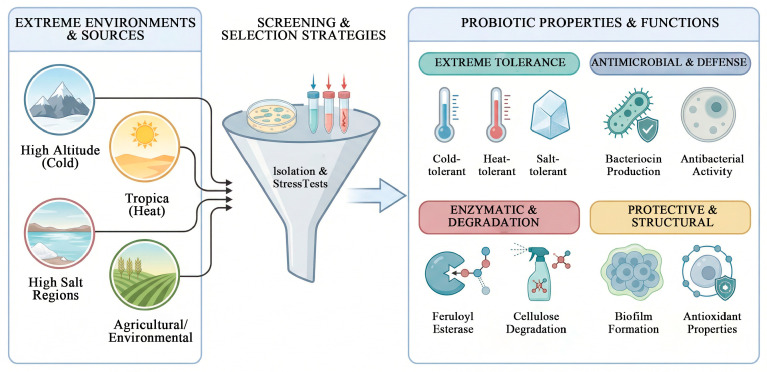
Probiotic properties of *lactic acid bacteria* (this figure was created with Figdraw.com, and the copyright code is ROAOU66646).

The role of LAB in degrading organophosphorus pesticides is attracting increasing attention. Relevant studies indicate that strains possessing such degradation capabilities can be isolated from diverse environments. For instance, Elanchezhian et al. [57] isolated LAB strains capable of degrading multiple organophosphorus pesticides from farm-produced probiotic preparations and transformed them into innovative technologies for controlling okra pests and degrading pesticides. [] Chi et al. [58] reported that inoculating *Lactiplantibacillus plantarum* during the low-temperature fermentation of pickles significantly accelerated the degradation of organophosphorus pesticides, reducing their half-life substantially from 111.8 weeks to a range of 26.8–37.3 weeks. Furthermore, Yuan et al. [59] found that the degradation capability of *Lactiplantibacillus plantarum CICC20261* against various organophosphorus pesticides was positively correlated with its phosphatase activity, demonstrating that the primary removal mechanism was enzymatic degradation rather than adsorption (Figure 3).

## 5. Omics Research Techniques for Silage Lactic Acid Bacteria

### 5.1. Microbiome Analysis

Microbiomics utilizes high-throughput sequencing and other technologies to analyze the composition, structure, and diversity of microbial communities in silage. Li et al. [2] employed 16S rRNA gene sequencing to accurately reveal the abundance changes and succession patterns of LAB genera (such as *Lactobacillus*, *Pediococcus*, *Leuconostoc*, *Weissella*, etc.) during fermentation by comparing samples treated with different fermentation durations, raw materials, or additives. In a study on sorghum silage, sequencing of the V3-V4 region of the 16S rRNA gene on the Illumina MiSeq platform further elucidated the effects of inoculating different lactic acid bacteria on bacterial diversity and community dynamics. Additionally, the combined treatment of natural grass silage with lactic acid bacteria and molasses has been shown to significantly increase the relative abundance of LAB, reduce overall microbial diversity, and effectively inhibit the growth of harmful microorganisms such as *Clostridia*, *Escherichia*, and *Enterobacter*, thereby improving silage fermentation quality.

### 5.2. Metabolomics Analysis

Metabolomics analysis of silage aims to systematically identify and quantify all small-molecule metabolites present, such as organic acids (e.g., lactic acid, acetic acid), alcohols, amino acids, sugars, and volatile flavor compounds. In a study investigating the effect of *Lactiplantibacillus plantarum* on whole-plant corn silage, Su et al. [60] utilized an integrated non-targeted metabolomics approach employing SPME-GC-MS, GC-TOF-MS, and LC-QE-MS/MS platforms. This comprehensive strategy led to the detection of 2087 metabolites, of which 1143 were reliably identified. Similarly, Fu et al. [61] applied UHPLC-MS/MS to analyze the metabolome of ryegrass silage under different cutting frequencies. Their findings revealed that differential metabolites were predominantly peptides and amino acids. Notably, KEGG enrichment analysis highlighted the significant contribution of biosynthetic pathways for arginine, alanine, aspartic acid, and glutamic acid. Furthermore, metabolomics serves as a valuable tool for validating the efficacy of candidate microbial strains. For instance, in rice straw silage, integrated bacterial community analysis and metabolomic results jointly confirmed *Limosilactobacillus fermentum Lac33* as a highly efficient fermentation starter [62].

### 5.3. Metagenomic Analysis

Metagenomics, by sequencing the genomic DNA of all microorganisms in silage, enables a comprehensive elucidation of the functional potential of microbial communities. The analytical approaches can be classified into two categories. The first is direct functional annotation based on sequencing data, which is exemplified by studies on dairy products from the Xilin Gol region. Through metagenomic sequencing combined with culturing methods, these studies not only revealed regional differences in lactic acid bacteria but also annotated a vast number of genes and metabolic pathways using the Uniprot database, identifying high concentrations of amino acid and sugar acid metabolism functions [63]. Similarly, analysis of high-temperature fermented Douchi (fermented soybean) clarified the activity of carbohydrate and amino acid metabolism during fermentation via the KEGG and CAZy databases [64]. The second is functional prediction based on marker gene sequences, such as in the work by Sun et al. [63], who utilized the PICRUSt2 tool to predict and compare pathways like carbohydrate metabolism and amino acid metabolism in silage bacterial communities. Thus, it reveals the metabolic potential of microorganisms under different fermentation conditions from a functional perspective. Together, these methods demonstrate the capability of metagenomics in uncovering the functional aspects of fermentation systems.

### 5.4. Transcriptomics Analysis

Transcriptomics captures and analyzes all transcribed RNA (primarily mRNA) within a silage microbial community at specific time points, directly reflecting the actively expressed functional genes. Due to the complex composition of silage materials, total RNA extraction is challenging. This process requires the removal of highly abundant rRNA to enrich mRNA, which is subsequently reverse-transcribed into cDNA libraries for sequencing. This technique has been applied to validate the contributions of specific functional bacterial strains. For instance, strain LR753, isolated from napier grass, was shown to significantly enhance the fermentation quality and aerobic stability of silage. This effect was confirmed through integrated metagenomic and metatranscriptomic analyses, which identified the key functional roles of LR753 during the fermentation process [65].

### 5.5. Applications and Challenges of Multi-Omics in the Production Process

**Figure 4:**
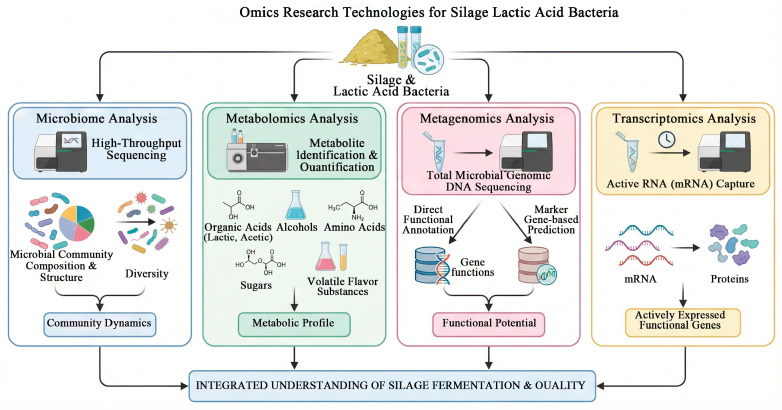
Omics studies in research on silage lactic acid bacteria (this figure was created with Figdraw.com, and the copyright code is RIWRWf449a).

Multi-omics technologies provide powerful tools for systematically analyzing the microbial mechanisms and metabolic networks of silage fermentation. Their advantage lies in the ability to comprehensively reveal the cascading regulatory processes from “gene–expression–protein–metabolite”, enabling precise prediction and control of the fermentation process. However, these technologies still face limitations such as high costs, complex data integration, environmental variability interference, and insufficient field-wide model applicability. Future challenges focus on developing low-cost in situ monitoring technologies, constructing integrated multi-omics predictive models, designing synthetic biology-based improved microbial inoculants, and promoting the translation of research outcomes into stable and efficient industrial applications to meet the demands of sustainable livestock development (Figure 4).

## 6. Benefits of Adding Lactic Acid Bacteria to Silage

### 6.1. Rapid Acidification and Reduced Nutrient Loss

The naturally occurring microbial community attached to plant surfaces is complex and includes harmful species. By introducing highly active exogenous homofermentative LAB (such as *Lactiplantibacillus plantarum*, *Pediococcus* spp., etc.), the fermentation process can be effectively guided. These lactic acid bacteria act as “pioneer strains”, rapidly utilizing soluble carbohydrates and producing substantial amounts of acid, thereby significantly lowering the pH of the silage within a short period. In studies on paper mulberry silage, the pH in treatment groups with added exogenous lactic acid bacteria was significantly lower than that in control groups [66]. Similarly, in oat silage, inoculation with *Lactobacillus rhamnosus* 753 significantly reduced the final pH to 3.95 after 60 days of ensiling, compared to 4.22 in the control group, demonstrating rapid and effective acidification [67]. In alfalfa silage, the addition of a novel composite lactic acid bacteria inoculant likewise effectively decreased pH [68]. This rapid acidification effectively suppresses the growth of spoilage bacteria, yeasts, and molds, minimizing protein degradation and dry matter losses, thereby preserving nutritional integrity.

### 6.2. Improving Fermentation Quality and Enhancing Palatability

High-quality LAB additives can steer the fermentation process toward the production of lactic acid as the primary metabolite, thereby avoiding butyric acid fermentation and excessive acetic acid accumulation. This effectively stimulates livestock appetite and increases feed intake. Li et al. [69] demonstrated that in natural pasture silage, the addition of LAB not only resulted in higher lactic acid content and lower pH, but also improved sensory quality.

### 6.3. Inhibition of Harmful Microorganisms and Ensuring Feed Safety

The synergistic effect of a low-pH environment and antimicrobial substances produced by LAB effectively suppresses the activity of undesirable microorganisms in silage, such as molds and yeasts. Research indicates that various LAB strains can synthesize bacteriocins with antimicrobial activity. For instance, *Lactobacillus plantarum* can produce several plantaricin bacteriocins, while *Pediococcus* species generate pediocins. These substances demonstrate significant inhibitory effects against common pathogens like *Listeria* and *Staphylococcus aureus*, as well as against certain spoilage-related clostridia and bacilli in silage [70]. Beyond bacteriocins, other antimicrobial strategies also prove effective. A study on maize-soybean silage showed that adding 1% clove (*Syzygium aromaticum* L.) increased aerobic stability from 34 h to over 168 h. Microbial community analysis revealed that clove treatment effectively inhibited the growth of spoilage fungi (Nakaseomyces and Pichia), which proliferated and became dominant (exceeding 70% relative abundance) in the control group after aerobic exposure, leading to pH rise and nutrient loss [71]. Furthermore, one study isolated a LAB strain from corn stover silage whose cell-free supernatant exhibited strong and stable antibacterial activity against *Salmonella*, *Micrococcus*, and *Escherichia coli*. This inhibitory activity remained stable under acidic conditions and after heat treatment, leading to the preliminary conclusion that the active component is a bacteriocin-like substance [72].

### 6.4. Degradation and Adsorption of Mycotoxins and Heavy Metals

Certain specific LAB strains, such as some *Lactiplantibacillus plantarum* and *Limosilactobacillus fermentum* strains, have been shown to adsorb or biodegrade common mycotoxins. The mechanisms of action include physical adsorption by the cell wall, enzymatic degradation, and metabolic transformation. Although the complete elimination of toxins in practical silage production remains challenging, this biodetoxification technology offers the potential for the safe utilization of feed ingredients with minor contamination, holding significant application prospects. Wang et al. [73] found that EPS-producing LAB strains generally exhibited superior performance in adsorbing heavy metals compared to non-EPS-producing strains. Mamoona Hameed et al. [74] reported that *Pediococcus pentosaceus* and *Limosilactobacillus fermentum* strains, isolated from human and animal feces, possessed a strong chelating capacity for Cd^2+^, removing 38% to 75% of cadmium from the culture medium after 24 h of incubation. Li et al. [75] investigated the process of Cd^2+^ removal from fruit juice by *Lactobacillus wei ZY-6 (Weizmannia coagulans ZY-6*), finding that its adsorption capacity was influenced by multiple factors and occurred primarily through the cell wall, leading to cell shrinkage post-adsorption. Mostafidi et al. [76] evaluated the adsorption performance of seven LAB strains for Cd^2+^, Pb^2+^, and Ni^2+^. Among them, Enterococcus faecalis exhibited the highest adsorption rates for these three metals, reaching 79.75%, 75.28%, and 83.99%, respectively. Yin et al. [77] used scanning electron microscopy (SEM) to observe significant shrinkage, depression, roughening, and adherent metal particles on the surface of LAB cells after heavy metal adsorption, confirming the accumulation of heavy metals on the cell surface. Fourier Transform Infrared (FTIR) spectroscopy analysis further indicated that functional groups on the cell wall, such as carboxyl, amino, hydroxyl, and phosphate groups, served as the primary sites involved in heavy metal binding.

### 6.5. Reducing Emissions of Methane and Other Gases

Research indicates that feeding Hanwoo steers with rye silage inoculated with *Lactobacillus plantarum* (1.5 × 10^10^ CFU/g) significantly altered ruminal fermentation, decreasing the acetate proportion (*p* = 0.044) and increasing the propionate proportion (*p* = 0.017). As methane production is directly linked to the acetate-to-propionate ratio, this metabolic shift underscores LAB’s potential to mitigate methane emissions by fundamentally modifying rumen fermentation pathways [78]. Further in vitro gas production tests confirmed that silage treated with specific lactic acid bacteria, such as *Lactobacillus plantarum* strains *AGR-1* and *AGR-2*, yielded significantly lower methane levels compared to the control. Moreover, the addition of *Lactobacillus plantarum* to *Stylosanthes* and rice straw silage not only reduced CO_2_ emissions but also decreased dry matter loss [79]. The underlying emission-reduction mechanisms may include: bacteriocins produced by lactic acid bacteria inhibiting the activity of hydrogen-producing bacteria in the rumen, or directly competing with methanogenic archaea for substrates, thereby interfering with methane production pathways [80].

### 6.6. Phyllosphere Endophytic and Epiphytic Lactic Acid Bacteria

In plant-based fermentation, naturally associated phyllosphere microbiota play a significant role, yet the composition and functions of endophytic and epiphytic bacteria remain to be fully elucidated. Zhou et al. [81] employed an integrated approach to investigate endophytic bacteria in alfalfa leaves. Their study revealed that alfalfa co-fermented with its native endophytes (EN group) exhibited significantly improved fermentation properties. Compared to the control, the EN group showed higher dry matter (DM), water-soluble carbohydrates (WSC), and crude protein (CP) contents (*p* < 0.05), alongside significantly lower pH, ammoniacal nitrogen (NH3-N), and acetic acid (AA), but higher lactic acid (LA) content (*p* < 0.05). This improvement was primarily due to the dominant growth of specific endophytic lactic acid bacteria (LAB), which also more effectively suppressed enterobacteria and yeasts than epiphytic strains. A key endophytic LAB, *Pediococcus pentosaceus EN5*, demonstrated superior carbohydrate utilization capability, attributed to unique genomic features such as specific enrichments in the mannose phosphotransferase system (Man-PTS) genes and carbohydrate-active enzymes (e.g., CBM35, GT32). This work demonstrates the considerable potential of endophytic LAB in promoting silage fermentation, offering a novel direction for developing targeted microbial inoculants. Ma et al. [82] further suggested that enriching or inducing beneficial endophytic lactic acid bacteria through regulation of plant growth conditions or breeding strategies could provide new approaches for creating silage inoculants.

### 6.7. Antimicrobial Peptides from Lactic Acid Bacteria

The application of antimicrobial peptides (AMPs) from lactic acid bacteria in food preservation is the most mature and widespread. Li et al. [83] found that the broad-spectrum antimicrobial peptide Bac-J23 can be used to preserve raw milk, effectively inhibiting microbial growth and spoilage at both 4 °C and 25 °C. With the growing accumulation of genomics, proteomics, and metabolomics data, artificial intelligence and machine learning are driving antimicrobial peptide research into a new phase. Researchers at the Institute of Chemical Physics, Chinese Academy of Sciences, have developed a novel deep learning-based screening method for dairy-derived antimicrobial peptides. As multi-omics data continues to expand, integrating big data algorithms to develop more accurate predictive models for antimicrobial peptide activity, structure-function relationship analysis, and toxicity prediction can significantly accelerate the computer-aided design and virtual screening of novel antimicrobial peptides.

### 6.8. Applications of Gene Editing Technology

With the advancement of gene editing technology, the application of the CRISPR-Cas system in lactic acid bacteria research is continuously deepening. Zhu et al. [84] have focused on identifying and utilizing endogenous CRISPR-Cas systems across a wider range of lactic acid bacterial species. These endogenous systems exhibit high compatibility with host cells, avoiding the toxicity associated with heterologous expression, thereby providing an effective tool for genetically recalcitrant strains. Yang et al. [85] have further introduced emerging technologies such as base editing, prime editing, and CRISPR-associated transposases (CAST) into lactic acid bacteria research. Among these, the CAST system enables precise integration of large DNA fragments without requiring DNA double-strand breaks or relying on homologous recombination, offering a breakthrough solution for metabolic engineering and genome reconstruction. The MetaEdit technology reported in Science [86] employs engineered bacteria to deliver CRISPR tools into the gut, It has been theoretically demonstrated that in situ genetic editing of specific bacteria within complex microbial communities is feasible. This provides a novel technical approach for the targeted modulation of silage microbial communities.

### 6.9. Utilization of Agricultural Waste

**Figure 5:**
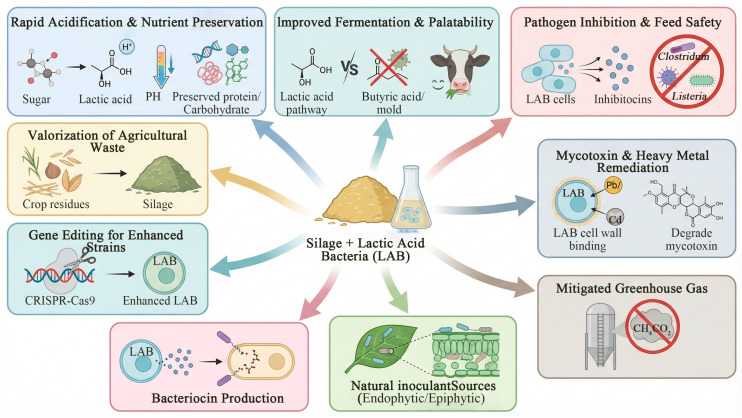
Advantages of selecting lactic acid bacteria for silage (this figure was created with Figdraw.com, and the copyright code is SWOST11ec2).

The importance of source reduction and resource utilization of agricultural waste provides specific guidance for the application of LAB technology in the treatment of straw, vegetable waste, and other similar materials. Li et al. [87] demonstrated that by co-ensiling pineapple residue with corn straw, the nutritional value of the pineapple residue was effectively preserved while improving the fermentation characteristics of the corn straw, resulting in palatable feed. Wu et al. [88] successfully preserved bioactive compounds such as chlorogenic acid and rutin in honeysuckle by-products (stems and leaves) through ensiling with specific LAB inoculants, highlighting their potential as functional feed additives. Guo et al. [3] found that certain lactic acid bacteria can degrade pesticide residues, offering a new approach for the safe utilization of contaminated straw or by-products. Fahim Ullah et al. [89] reported that prickly ash seed meal contains irritating alkaloids and alkylamides that limit its feed value. After solid-state fermentation with lactic acid bacteria, these toxic components were degraded by 39.01% and 50.41%, respectively, accompanied by a significant decrease in pH, providing a green solution for the safe conversion of this by-product into feed (Figure 5).

## 7. Limitations of Adding Lactic Acid Bacteria to Silage Feed

Although lactic acid bacteria additives are considered effective microbial preparations for improving silage quality, their application also has multiple limitations. Firstly, the effectiveness of lactic acid bacteria inoculation essentially depends on the adaptability of the strain to specific forage, manifested as significant differences in the interaction between the strain and the crop. For example, some strains of *Lactobacillus plantarum* have excellent fermentation performance in high sugar corn, but may not be effective in low sugar and high protein alfalfa [90]. Secondly, the effectiveness of lactic acid bacteria additives depends on the initial conditions of the raw materials. For example, when the soluble carbohydrates in the raw materials are insufficient or the moisture content is not suitable, the inoculation effect will significantly decrease. Thirdly, the function of bacterial strains is singular; homogenous fermentation bacteria that rapidly produce acid cannot solve the problem of aerobic decay after opening, while heterologous fermentation bacteria that can improve stability have the disadvantages of low fermentation efficiency and significant dry matter loss. In actual production, cost and operation are also important limiting factors, including the purchase cost of microbial agents, equipment requirements for uniform spraying, and storage issues of live bacterial preparations. In addition, the protective effect of lactic acid bacteria on proteins is limited, and they are basically unable to degrade lignin to improve fiber digestibility [91]. Finally, improper use of lactic acid bacteria additives may also pose potential risks, such as accelerating aerobic decay or producing substances that affect palatability. And lactic acid bacteria cannot replace good field and cellar storage management. It can only serve as a “fermentation regulator” based on high-quality feed, and cannot compensate for fundamental defects such as late harvesting of raw materials or inadequate compaction and sealing. In summary, the prerequisite for its successful application is to fully recognize these limitations and consider vaccination technology as a systematic measure that must be integrated with raw material management, compaction and sealing, and retrieval management.

## 8. Conclusions

**Figure 6:**
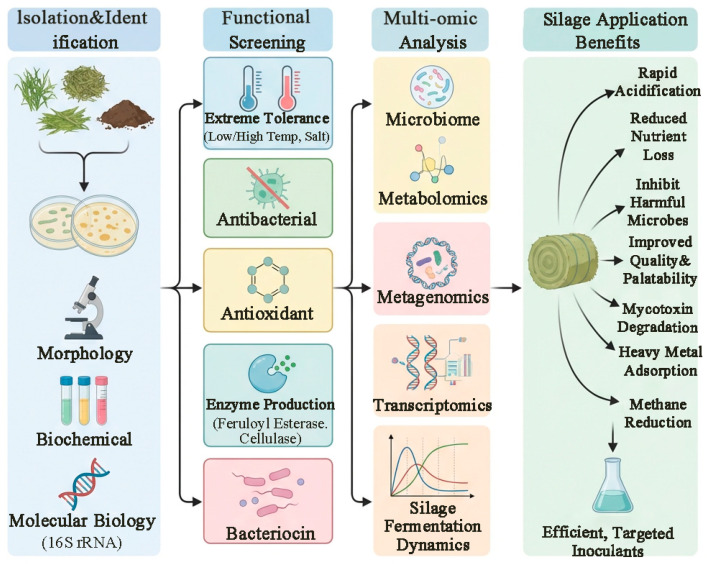
Flowchart of the screening, isolation, and identification of lactic acid bacteria (this figure was created with Figdraw.com, and the copyright code is OSAOY3f1c3).

The screening of lactic acid bacteria has evolved from traditional phenotypic identification to an integrated approach that combines molecular biology with multi-omics technologies. This advancement allows for the efficient isolation of superior strains with desirable traits-such as cold resistance, heat resistance, salt tolerance, antioxidant activity, and bacteriocin production-from extreme environments and various fermented foods. Functional lactic acid bacteria not only rapidly initiate silage acidification, inhibit harmful microorganisms, reduce nutrient loss, and improve the fermentation quality and palatability of feed, but also demonstrate significant potential in degrading mycotoxins, adsorbing heavy metals, and reducing methane emissions. The application of microbiomics, metabolomics, metagenomics, and transcriptomics has further revealed the central role of lactic acid bacteria in the silage micro-ecosystem and their interaction mechanisms. Future efforts should focus on exploring phyllosphere endophytic and epiphytic microbial resources, studying plant-microbe interactions, promoting the conversion of patented strains into practical inoculants, and achieving precise regulation of the silage process through deep integration of multi-omics and conventional techniques. This will facilitate the selection and breeding of a new generation of lactic acid bacterial agents, contributing to the development of green animal husbandry (Figure 6).

## Data Availability

No new data were created or analyzed in this study.

## Abbreviations

The following abbreviations are used in this manuscript:

LAB	Lactic acid bacteria
WSC	Water-soluble carbohydrate
EPS	Exopolysaccharides
MRS	De Man, Rogosa, and Sharpe
FJLB	Fermented juice of tropical crops
HPLC	High-performance liquid chromatography
SSF	Simultaneous saccharification and fermentation
SEM	Scanning electron microscopy
FTIR	Fourier transform infrared
Man-PTS	Mannose phosphotransferase system
AMPs	Antimicrobial peptides
CAST	CRISPR-associated transposases

## References

[1] Yan F., Zhou H. (2016). Overviews and Applications of the CRISPR/Cas9 System in Plant Functional Genomics and Creation of New Plant Germplasm. Sci. China Life Sci..

[2] Li Y., Du S., Sun L., Cheng Q., Hao J., Lu Q., Ge G., Wang Z., Jia Y. (2022). Effects of Lactic Acid Bacteria and Molasses Additives on Dynamic Fermentation Quality and Microbial Community of Native Grass Silage. Front. Microbiol..

[3] Guo X., Xu D., Li F., Bai J., Su R. (2023). Current Approaches on the Roles of Lactic Acid Bacteria in Crop Silage. Microb. Biotechnol..

[4] Okoye C.O., Wang Y., Gao L., Li X., Sun J., Jiang J. (2023). The Performance of Lactic Acid Bacteria in Silage Production: A Review of Modern Biotechnology for Silage Improvement. Microbiol. Res..

[5] Sarita B., Samadhan D., Hassan M.Z., Kovaleva E.G. (2025). A comprehensive review of probiotics and human health-current prospective and applications. Front. Microbiol..

[6] Azevedo M.D. (2013). Engineering of Lactic Acid Bacteria Strains Modulating Immune Response for Vaccination and Delivery of Therapeutics. Ph.D. Thesis.

[7] Zhang F., Ni L., Zhang Z., Luo X., Wang X., Zhou W., Chen J., Liu J., Qu Y., Liu K. (2024). Recombinant *L. lactis* Vaccine LL-plSAM-WAE Targeting Four Virulence Factors Provides Mucosal Immunity Against *H. pylori* Infection. Microb. Cell Factories.

[8] Van Pijkeren J.P., Britton R.A. (2014). Precision Genome Engineering in Lactic Acid Bacteria. Microb. Cell Factories.

[9] Yue B., Sun F., Fu D., Yang Z., Zhang F. (2022). Effects of homo/heterotypic lactic acid bacteria on nutritional characteristics of whole plant corn silage. Heilongjiang Anim. Sci. Vet. Med..

[10] Duan Y., Tan C., Wang Y., Qin G., Huo Y., Cai Y. (2008). The Characteristics of Microorganisms and Lactic Acid Bacteria in Silage. Henan Agric. Sci..

[11] Yang J., Zhang L., Jiang H., He Z. (2015). Isolation and Identification of Lactic Acid Bacteria in Pickled Vegetables from Meishan City. Food Sci..

[12] Liu T., Pan D. (2011). Screening of Lactic Acid Bacteria with Antioxidant Activity. Food Sci..

[13] Peterkin P.I., Vanderzant C., Splittstoesser D.F. (1992). Compendium of Methods for the Microbiological Examination of Foods.

[14] Lv Y. (2017). Selection and Identification of Acid- and Bile-Resistant Probiotic Lactic Acid Bacteria. Food Mach..

[15] Hammes W.P., Vogel R.F., Wood B.J.B., Holzapfel W.H. (1995). The genus *Lactobacillus*. The Genera of Lactic Acid Bacteria.

[16] Qi J., Wang H. (2020). Isolation and Identification of Lactic Acid Bacteria from Natural Fermented Sauce of Pickle and Study of Their Biological Characteristics. Acad. Period. Farm Prod. Process..

[17] Niu M., Wang K., Li B. (2018). Research Progress of Exopolysaccharide from Lactic Acid Bacteria. J. Heilongjiang Bayi Agric. Univ..

[18] El Ahmadi K., Haboubi K., El Allaoui H., El Hammoudani Y., Bouhrim M., Eto B., Shahat A.A., Herqash R.N. (2025). Isolation and preliminary screening of lactic acid bacteria for antimicrobial potential from raw milk. Front. Microbiol..

[19] Xie F., Zhang F., Zhou K., Zhao Y., Zhao Q., Sun H. (2017). Isolation, Identification and Fermentation Optimization of Lactic Acid Bacteria for Aquaculture Water Purification. Acta Microbiol. Sin..

[20] Duan X., Qiqirilige, Qiu C., Lai M., Wuyundalai (2020). Screening, identification, purification and characterization of alkaline phosphatase from *Lactobacillus*. Microbiology.

[21] Hu C., Yang X., Guo Q., Li B., Zheng Y., Huang H., Fan Y. (2023). Isolation, identification, and fermentation characteristics of lactic acid bacteria from the radish pickles brines. Food Ferment. Ind..

[22] Waroka E., Fachrial E., Lister I.N.E., Kurnia M.A. (2023). Antibacterial Activity and 16S Rrna Gene Sequencing of Lactic Acid Bacteria from Homemade Fermented Milk in Medan, Indonesia. J. Health Sci..

[23] Ilyanie Y., Faujan N.H., Muryany M.Y.I. (2023). Species Identification of Potential Probiotic Lactic Acid Bacteria Isolated from Malaysian Fermented Food Based on 16S Ribosomal RNA (16S Rrna) and Internal Transcribed Spacer (ITS) Sequences. Malays. Appl. Biol..

[24] Putra R.W., Fevria R. (2021). Isolation and Identification of Probiotic Candidate Lactic Acid Bacteria (lab) from Shrimp Paste (*mysis Relicta*) Based on 16s Rrna Gene. Bioscience.

[25] Sengun I.Y., Yalcin H.T., Kilic G., Ozturk B., Peker A.K., Terzi Y., Atlama K. (2024). Identification of Lactic Acid Bacteria Found in Traditional Shalgam Juice Using 16S Rrna Sequencing and Evaluation of Their Probiotic Potential In Vitro. Food Biosci..

[26] Yang X.D., Yuan X.J., Guo G., Cui Z.M., Li J.F., Bai X., Ba S., Shao T. (2015). Isolation and identification of low temperature-tolerant lactic acid bacteria from legume silages in Tibet. Acta Prataculturae Sin..

[27] Lin D., Ju Z., Cai J., Zhao G. (2022). Screening and Identification of Cold-Tolerant Lactic Acid Bacteria Attached to Oats from the Qinghai-Tibet Plateau. Acta Prataculturae Sin..

[28] Chen L., Wei B., Zheng L., Zhang Y., Yan X., Yu W. (2020). Screening of Low-Temperature Tolerant Lactic Acid Bacteria from the Soil of the Pristine Forests of Changbai Mountain. China Anim. Husb. Vet. Med..

[29] Xu D., Cao L., Jing P., Zhang Y., Zhang H., Zhang W., Guo X. (2017). Screening of Cryophilic Lacto-Bacteria from Several Qinghai-Tibet Plateau Herbages. J. Microbiol..

[30] Liu Y., Zi X., Chen T., Sun R., Li M., Tang K. (2022). Isolation, Identification, and Screening of Lactic Acid Bacteria in Natural Forage Silage from Hainan. J. Grassl..

[31] Pitiwittayakul N., Bureenok S., Schonewille J.T. (2021). Selective Thermotolerant Lactic Acid Bacteria Isolated from Fermented Juice of Epiphytic Lactic Acid Bacteria and Their Effects on Fermentation Quality of Stylo Silages. Front. Microbiol..

[32] Papadopoulou E., Rodriguez De Evgrafov M.C., Kalea A., Tsapekos P., Angelidaki I. (2023). Adaptive laboratory evolution to hypersaline conditions of lactic acid bacteria isolated from seaweed. New Biotechnol..

[33] He X., Zhao L., Li M., Zhou X., Che L., Liu T. (2022). Screening of Salt-Tolerant Lactic Acid Bacteria and Research on Their Mutagenesis Breeding and Tolerance. Food Ferment. Ind..

[34] Sun H., Tang H., Liu H., Cui Z., Pan S., Hou X., Yang J. (2023). Screening of acid and salt tolerant bacteriocin-producing lactic acid bacteria in brine. China Feed.

[35] Wang C., Liu D., Zhai G., Niu X., He R., Dong G., Liu Y. (2022). Selection and Identification of Lactic Acid Bacteria for Silage Fermentation from Native Sources. Shandong Anim. Husb. Vet. Med..

[36] Xiao Z., Zhong R., Lu W., Zhang F., Lin L. (2023). Screening and Identification of Lactic Acid Bacteria with Antimicrobial Activity in Fruit Ferments. Mod. Food Sci. Technol..

[37] Lu Y., Zhang N., Ou S., Hu X. (2013). Screening of *Lactobacillus* Producing Ferulic Acid Esterase and Gene Cloning. Food Ind. Technol..

[38] Xu Z., He H., Zhang S., Guo T., Kong J. (2017). Characterization of Feruloyl Esterases Produced by the Four *Lactobacillus* Species: *L. amylovorus*, *L. acidophilus*, *L. farciminis* and *L. fermentum*, Isolated from Ensiled Corn Stover. Front. Microbiol..

[39] Chen J., Pang H., Wang L., Ma C., Wu G., Liu Y., Guan Y., Zhang M., Qin G., Tan Z. (2022). Bacteriocin-Producing Lactic Acid Bacteria Strains with Antimicrobial Activity Screened from Bamei Pig Feces. Foods.

[40] Li Y., Wu Y., Peng Z., Long L., Guo Q., Tian L., He Z., Xiang S., Kang Y., Guan T. (2024). Isolation and identification of bacteriocin-producing lactic acid bacteria from Daqu and mining of bacteriocin gene. Biologia.

[41] Yi L., Qi T., Hong Y., Deng L., Zeng K. (2020). Screening of bacteriocin-producing lactic acid bacteria in Chinese homemade pickle and dry-cured meat, and bacteriocin identification by genome sequencing. LWT.

[42] Meruvu H., Harsa S. (2022). Lactic acid bacteria: Isolation–characterization approaches and industrial applications. Crit. Rev. Food Sci..

[43] Lv L., Yang X., Wang A., Zhong X., Huang G., Zhao J. (2022). Screening of Lactic Acid Bacteria with Antibacterial Activity in Soy Sauce Residue and Its Antibacterial Properties. Sci. Technol. Food Ind..

[44] Zhang L., Ding Y., Pan Y., Li K., Geng Y., Zhou J., Xu H., Li H., Xu Z., Shi J. (2023). Screening of bacteriocin-producing lactic acid bacteria and the probiotic properties evaluation. Food Ferment. Ind..

[45] Weinbrenner D.R., Barefoot S.F., Grinstead D.A. (1997). Inhibition of Yogurt Starter Cultures by Jenseniin G, a Propionibacterium Bacteriocin1. J Dairy Sci..

[46] Hu Y., Zhao Y., Jia X., Liu D., Huang X., Wang C., Zhu Y., Yue C., Deng S., Lyu Y. (2023). Lactic acid bacteria with a strong antioxidant function isolated from “Jiangshui”, pickles, and feces. Front. Microbiol..

[47] Wang J., Liu Y., Zheng H., Xin J., Zhong Z., Liu H., Huang Y., Fu H., Zhou Z., Peng G. (2024). Screening and Genome Analysis of Heat-Resistant and Antioxidant Lactic Acid Bacteria from Holstein Cow Milk. Front. Microbiol..

[48] Wang J., Liu P., Xiao H. (2022). Physicochemical and Antioxidant Characteristics of Rice Bean Milk Fermented by Functional Lactic Acid Bacteria. Food Sci. Technol..

[49] Yang C., Huang B., Lin J., Yang Q., Guo Y., Liu D., Sun B. (2024). Isolation and screening of high biofilm producing lactic acid bacteria, and exploration of its effects on the microbial hazard in corn straw silage. J. Hazard. Mater..

[50] Zhang Y., He Y., Gu Y., Wang Y., Zheng Y. (2021). High Biofilm-Forming Lactic Acid Bacteria: Stress Resistance and Antioxidant Properties. Trans. Chin. Soc. Agric. Eng..

[51] Olszewska M.A., Nynca A., Białobrzewski I. (2019). Biofilm formation by lactobacilli and resistance to stress treatments. Int. J. Food Sci. Technol..

[52] Li F., Jia M., Chen H., Chen M., Su R., Usman S., Ding Z., Hao L., Franco M., Guo X. (2024). Responses of microbial community composition and CAZymegene enrichment in ensiled Elymus nutans to altitudinal gradients in alpine region. Appl. Environ. Microb..

[53] Li M., Zi X., Lv R., Hu H., Tang J., Zhou H. (2020). Effects of Adding Lactic Acid Bacteria and Cellulase on the Silage Quality and Rumen Degradability of King Grass. Chin. J. Anim. Sci..

[54] Chen C., Xin Y., Li X., Ni H., Zeng T., Du Z., Guan H., Wu Y., Yang W., Cai Y. (2022). Effects of Acremonium Cellulase and Heat-Resistant Lactic Acid Bacteria on Lignocellulose Degradation, Fermentation Quality, and Microbial Community Structure of Hybrid Elephant Grass Silage in Humid and Hot Areas. Front. Microbiol..

[55] Usman S., Zhang Y., Yang X., Guo X., Shen Y. (2025). Lignocellulose Degradation, Enzymatic Saccharification and Bioethanol Production from Whole-Crop Sweet Sorghum Silage Inoculated with Feruloyl-Esterase Producing Lactic Acid Bacteria. Int. J. Biol. Macromol..

[56] Berlowska J., Cieciura W., Borowski S., Dudkiewicz M., Binczarski M., Witonska I., Otlewska A., Kregiel D. (2016). Simultaneous Saccharification and Fermentation of Sugar Beet Pulp with Mixed Bacterial Cultures for Lactic Acid and Propylene Glycol Production. Molecules.

[57] Elanchezhyan K., Rajinimala N., Raja D.L., Gokila A.S.S. (2024). Farm-made pesticides-degrading lactic acid bacterial formulation for pest management in okra, *Abelmoschus Esculentus* (L.) Moench. Appl. Ecol. Environ. Res..

[58] Chi T., Fang J., Zheng Y., Liu P. (2015). Effect of Lactic acid bacteria on the degradation of organophosphorus pesticides at low temperature in pickled Chinese cabbage. China Dairy Ind..

[59] Yuan S., Li C., Yu H., Xie Y., Guo Y., Yao W. (2021). Screening of Lactic Acid Bacteria for Degrading Organophosphorus Pesticides and Their Potential Protective Effects Against Pesticide Toxicity. LWT-Food Sci. Technol..

[60] Su R., Ke W., Bai J., Wang M., Usman S., Xie D., Xu D., Chen M., Guo X. (2023). Comprehensive Profiling of the Metabolome in Corn Silage Inoculated with or Without Lactiplantibacillus Plantarum Using Different Untargeted Metabolomics Analyses. Arch. Fur Tierernahr..

[61] Fu Z., Sun L., Hou M., Hao J., Lu Q., Liu T., Ren X., Jia Y., Wang Z., Ge G. (2022). Effects of Different Harvest Frequencies on Microbial Community and Metabolomic Properties of Annual Ryegrass Silage. Front. Microbiol..

[62] Sun Y., Sun Q., Tang Y., Li Q., Tian C., Sun H. (2023). Integrated microbiology and metabolomic analysis reveal the improvement of rice straw silage quality by inoculation of *Lactobacillus* brevis. Biotechnol. Biofuels Bioprod..

[63] Sun J., Su Q., Du P., Xia Y., Zhao J., Yu J., Chen Y. (2023). Isolation and Identification of Lactic Acid Bacteria and Microbial Diversity in Fresh Cow Milk from Xilin Gol. Food Sci..

[64] Xian F., Zhao W., Wang S., Li H., Yang H., Wang Y. (2023). Analysis of the Community Structure and Functional Annotation of High-Temperature Rapid Fermented Douchi Based on Metagenomic Sequencing Technology. Food Ind. Technol..

[65] Guan H., Shuai Y., Ran Q., Yan Y., Wang X., Li D., Cai Y., Zhang X. (2020). The microbiome and metabolome of Napier grass silages prepared with screened lactic acid bacteria during ensiling and aerobic exposure. Anim. Feed Sci. Technol..

[66] Shu G., Wang H., Zhang S., Zhang Y., Zhang L., Cheng J., Fan C., Chen L. (2023). Effect of lactic acid bacteria from Broussonetiapapyrifera on silage fermentation. Pratacultural Sci..

[67] Tahir M., Li J., Xin Y., Wang T., Chen C., Zhong Y., Zhang L., Liu H., He Y., Wen X. (2023). Response of Fermentation Quality and Microbial Community of Oat Silage to Homofermentative Lactic Acid Bacteria Inoculation. Front. Microbiol..

[68] Dong W., Zhao S., Zhang L., Liu J., Liu J., Zhang J., Kou J., Yang W. (2023). Effects of the Addition of a Novel Compound Lactic Acid Bacteria on Alfalfa Silage Quality and Bacterial Community Composition. Chin. J. Grassl..

[69] Li X., Guan H., Shuai Y., Li X., Peng A., Li C., Pu Q., Yan Y., Zhang X. (2019). Effects of single and multiple inoculants on Hemarthria compressa silage quality. Acta Prataculturae Sin..

[70] Zhang J., Xie Y., Jin J., Duan H., Liu H., Zhang H. (2016). Screening of bacteriocin-producing *Lactobacillus plantarum* and study on antimicrobial characteristics of bacteriocin. Food Ind. Technol..

[71] Li X., Jing X., Zheng H., Du M., Wu Q., Yang W., Yan Y. (2025). [SI_Forage]_Decoding the microbial community and quorum sensing of whole plant maize-soybean silage under strip intercropping systems and techniques for improving ensiling quality. J. Integr. Agr..

[72] Li D., Ni K., Pang H., Wang Y., Jin Q. (2015). Identification and Antimicrobial Activity Detection of Lactic Acid Bacteria Isolated from Corn Stover Silage. Asian Australas. J. Anim. Sci..

[73] Wang Y., Han J., Ren Q., Liu Z., Zhang X., Wu Z. (2023). The Involvement of Lactic Acid Bacteria and Their Exopolysaccharides in the Biosorption and Detoxication of Heavy Metals in the Gut. Biol. Trace Elem. Res..

[74] Hameed M., Shakir H.A., Mushtaq M., Gul Z., Javed S., Arshad N. (2024). A Comparative Study on Heavy Metal (cadmium) Tolerance and Biosorption Capacity of Locally Isolated Lactic Acid Bacteria. Bioremediation J..

[75] Li W., Chen Y., Wang T. (2020). Cadmium Biosorption by Lactic Acid Bacteria Weissella Viridescens ZY-6. Food Control.

[76] Mostafidi M., Sanjabi M.R., Mojgani N., Eskandari S., Bidgoli S.A. (2024). Assessment of Cadmium, Lead and Nickel Removal Capacity of Lactic Acid Bacteria from Aqueous Solutions and Fresh Edible Vegetables. Iran. Food Sci. Technol. Res. J..

[77] Yin R. (2016). Mechanistic Analysis of *Lactobacillus plantarum* CCFM8661 in Adsorbing Lead Ions and Alleviating Lead Toxicity in Intestinal Cells. Master’s Thesis.

[78] Choi Y., Kim J., Bang G., Kim N., Thirugnanasambantham K., Lee S., Kim K.H., Bharanidharan R. (2024). Effect of Sodium Formate and Lactic Acid Bacteria Treated Rye Silage on Methane Yield and Energy Balance in Hanwoo Steers. Peerj.

[79] Shen C., Chen M., Li G., Zhang J. (2012). Effects of Adding Lactic Acid Bacteria and Pineapple Peel on the Silage Quality of Pennisetum purpureum. J. Grassl. Sci..

[80] Jin Y. (2012). The Effect of Inhibiting Rumen Fungi on Microbial Fermentation and Methane Emissions in Goats. Master’s Thesis.

[81] Zhou H., Jia S., Gao Y., Li X., Lin Y., Yang F., Ni K. (2024). Characterization of Phyllosphere Endophytic Lactic Acid Bacteria Reveals a Potential Novel Route to Enhance Silage Fermentation Quality. Commun. Biol..

[82] Ma T., Xin Y., Chen X., Wen X., Wang F., Liu H., Zhu L., Li X., You M., Yan Y. (2025). Effects of Compound Lactic Acid Bacteria Additives on the Quality of Oat and Common Vetch Silage in the Northwest Sichuan Plateau. Fermentation.

[83] Li D., Zhou Y. (2017). Current Status and Research Progress of Animal-Derived Lactic Acid Bacteria. Agric. Prod. Process..

[84] Zhu Q., Xu C., Zhang S., Xie N., Pang X., Lü J. (2022). Advances in Utilizing the Endogenous CRISPR-Cas System for Genome Editing of Lactic Acid Bacteria. Sheng Wu Gong Cheng Xue Bao Chin. J. Biotechnol..

[85] Yang Y., Ai L.Z., Xiong Z.Q., Yang Y.J., Song X. (2022). Research Progress of Application of Transposon Editing Technique and CRISPR Editing Technique in Directional Improvement of Lactic Acid Bacteria. J. Food Sci. Technol..

[86] Gelsinger D.R., Ronda C., Ma J., Kar O.B., Edwards M., Huang Y., Mavros C.F., Sun Y., Perdue T., Vo P.L. (2025). Metagenomic editing of commensal bacteria in vivo using CRISPR-associated transposases. Science.

[87] Li C. (2024). Effects of Lactic Acid Bacteria on the Silage Quality of Pineapple Residue and Corn Straw Mix and In Vitro Rumen Fermentation Characteristics in Buffalo. Master’s Thesis.

[88] Wu Y., Xiao Y., Okoye C.O., Gao L., Chen X., Wang Y., Jiang J. (2025). Fermentation Profile and Bioactive Component Retention in Honeysuckle Residue Silages Inoculated with Lactic Acid Bacteria: A Promising Feed Additive for Sustainable Agriculture. Ind. Crop. Prod..

[89] Ullah F., Baichuan W., Yongjun Z., Rahman S.U., Assefa M., Shah T.A., Elossaily G.M., Almohammed O.A. (2025). Degradation of alkaloids and alkylamides in Zanthoxylum bungeanum meal by lactic acid bacteria via solid-state fermentation. Amb. Express.

[90] Tahir M., Wang T., Zhang J., Xia T., Deng X., Cao X., Zhong J. (2025). Compound Lactic Acid Bacteria Enhance the Aerobic Stability of Sesbania Cannabina and Corn Mixed Silage. BMC Microbiol..

[91] Irawan A., Sofyan A., Ridwan R., Abu Hassim H., Respati A.N., Wardani W.W., Sadarman, Astuti W.D., Jayanegara A. (2021). Effects of Different Lactic Acid Bacteria Groups and Fibrolytic Enzymes As Additives on Silage Quality: A Meta-Analysis. Bioresour. Technol. Rep..

